# Assessment of the Health-Related Behaviors of Men Practicing Combat Sports and Martial Arts

**Published:** 2019-10

**Authors:** Dariusz BOGUSZEWSKI, Jakub Grzegorz ADAMCZYK, Dariusz BIALOSZEWSKI

**Affiliations:** 1. Department of Rehabilitation, Physiotherapy Division, 2nd Faculty of Medicine, Medical University of Warsaw, Warsaw, Poland; 2. Department of Theory of Sport, Józef Pilsudski University of Physical Education in Warsaw, Warsaw, Poland

**Keywords:** Lifestyle, Judo, Aikido, Taekwondo, Physical activity

## Abstract

**Background:**

Physical activeness is one of the main elements of lifestyle in terms of preventing civilization diseases. The main purpose of this study was to assess the selected health behaviors of men practicing combat sports and martial arts and to find out what features or variables may have an influence one's lifestyle.

**Methods:**

Overall, 561 men aged 17–35 yr were enrolled and divided into three groups. Studies were conducted from March 2011 to June 2015 in Warsaw, Poland. The main research tool was Juczyński's Health Behaviour Inventory. The questionnaire includes 24 statements – descriptions of various health-related attitudes and behaviours – divided into four categories: proper eating habits, preventive behaviours, positive psychical attitude, and health practices.

**Results:**

The persons practicing combat sports and martial arts presented the highest level of health behaviours. The result of Group 1 was significantly higher than the result of Group 2 (*P*=0.05) and Group 3 (*P*<0.001). The sportsmen from Group 1 obtained the highest result in the category of psychical attitude (3.5), which was similar to the result of the non-active group (3.29). The men belonging to Group 2 rated highest their eating habits (3.37). All groups obtained the lowest results in the category of preventive behaviours.

**Conclusion:**

The men practicing combat sports and martial arts showed high general level of health behaviours. It may give evidence to the intellectualization of sport and health training process, as well as to the fact of adopting the desired behaviours in everyday life.

## Introduction

The problem of civilization diseases affects increasingly more people. Lifestyle plays a particularly important part of their prevention. It concerns especially those diseases which develop as a result of inappropriate habits. Cardiovascular diseases constitute the biggest and the most dangerous group. Their root causes are the simplest health-related behaviours, such as the quality and amount of consumed food, the use of stimulants (cigarettes, alcohol), or the level of physical activeness and fitness (1). The first symptom may be the increased body weight. In the developed countries the percentage of overweight and obese persons exceeds 50% of population (2). Physically active persons are more often aware of the importance of lifestyle in keeping good health, which is why they maintain healthier diet and less frequently use stimulants. Besides, physical endurance constitutes one of the indicators of human's health. (3, 4). This dependency does not equally concern the persons practicing different forms of physical activity or sports disciplines.

Walking is the simplest form of health training. This popular form of aerobic training allows controlling the effort as appropriate to the age and capacity of an exercising person. The same amount of calories is consumed during a fast walk as during running the same distance. Biking may be another form of activity. It ensures the protection of ankle, knee and hip joints from harmful stress. Another beneficial form of exercise is swimming or exercising in water (especially recommended to obese persons). The main advantages of water-based environment include reducing body weight with simultaneous generating resistance during exercising. Besides, exercising in water allows for working out multiple muscle groups at the same time. Moving in a water-based environment reduces the sensation of pain or discomfort that appears during exercising (5, 6).

Combat sports and martial arts are less often recognized as one of the forms of health training. Practicing combat sports and martial arts, besides improving physical fitness, shapes volitional traits, ethics, and personality as well (7, 8). The educational aspect is somewhat inscribed in the sports training system (9).

The main purpose of this study was to assess the selected health behaviours of men practicing combat sports and martial arts and to present the features or variables that may influence one's lifestyle.

## Materials and Methods

The study covered 561 persons aged 17–35. Studies were conducted from March 2011 to June 2015 in Warsaw, Poland. Group 1 – the study group, consisted of 221 men practicing selected combat sports and martial arts (judo, aikido, capoeira, taekwondo, kickboxing, and wrestling). All the persons practicing taekwondo and wrestling, and 63 judokas and 22 kickboxers are sportsmen possessing a license of Polish sport associations and taking part in championships. The control groups (n=340) consisted of physically active persons (Group 2, n=160) who practice (competitive) athletics and powerlifting, and (amateur) bodybuilding and dancing, as well as persons not engaged in any kind of organized and regular physical activities (Group 3, n=180). The groups did not differ significantly in terms of age, body weight and height (Table 1).

**Table 1:** Biometric characteristic of examined people

***Groups***	***Number of people [n]***	***Age [yr]***	***Body mass [kg]***	***Body heigth [cm]***	***BMI [kg/m^2^]***
Group 1 – combat sports	221	22.21±4.50	76.11±11.81	177.07 ±6.98	24.21 ±3.02
Group 2 – active	160	22.67±5.33	80.90±15.27	180.07 ±7.60	24.86 ±3.98
Group 3 – non-active	180	22.84±4.40	79.91±12.35	180.66 ±7.41	24.43 ±3.10

Additionally, for the purpose of comparison, the studied persons practicing combat sports and martial arts were divided into subgroups based on the criterion of sport discipline and age (junior/youth – senior), training experience (up to 9 yr – more than 10 yr) and frequency of training (up to 3 times a week – more than 4 times a week).

The research tool was Juczyński's Health Behaviour Inventory (10). The questionnaire includes 24 statements, which are descriptions of various health-related attitudes and behaviours. The studied persons indicate the frequency on the basis of a five-grade rating system: 1-almost never, 2-seldom, 3-from time to time, 4-often, 5-almost always. The questions concern the four basic categories of health behaviours:

- Proper nutrition habits (the kind and quality of consumed food) – NH,
- Preventing behaviours (complying with the principles of preventing civilization diseases, possessing and obtaining information on one's health and disease) – PB,
- Positive psychical attitude (avoiding negative emotions, tension, and stress) – PA,
- Health practices (everyday habits such as being physically active, sleep, recreation) – HP (10).

The marked answers (numeral values) are summed up in order to obtain the general Health Behaviour Indicator – HRB. Moreover, the intensity of the four categories of health behaviours mentioned above is calculated by providing the average result. The obtained overall result allows for conventional classification of the studied persons as those of the low 24–77 points, average 78–91 or high 92–120 level of health behaviours (10, 11).

In order to establish the applied statistical tools, Kolmogorov-Smirnov test was investigated whether the variable dependent normal distribution. All results indicate incompatibility with normal distribution, use the following nonparametric tests. The differences between Group 1 (Combat sports), and 2 (Active) and 3 (Non-active) were measured by the means of the Mann-Whitney U test. The differences between the many groups (particular disciplines) were established based on Kruskal-Wallis test. The relationships between particular variables (e.g., age and health behaviours, or training experience) were established by the means of the Spearman's rho correlation analysis. The minimal statistical significance was set at *P*≤0.05.

Non-invasive studies do not require the consent of the Medical University of Warsaw's Bioethics Committee.

## Results

The persons practicing combat sports and martial arts presented the highest level of health behaviours (expressed in health behaviour indicator). The average total result of Group 1 was significantly higher than the result of Group 2 (*P*=0.05) and Group 3 (*P*<0.001). The persons who are not physically active obtained a significantly lower result than the representatives of active groups (Table 2).

**Table 2:** Health-related behaviours of examined people

***Groups***	***Nutrition habits***	***Prophylactic behaviour***	***Positive attitude***	***Healthy practices***	***Sum***
Group 1 – combat sports	3.37 ±0.75	3.24 ±0.81	3.50 ±0.71	3.46 ±0.62	81.39 ±12.99
Group 2 – active	3.37 ±0.84	3.06 ±0.72	3.30 ±0.69	3.36 ±0.73	78.53 ±13.57
Group 3 – non-active	3.19 ±0.87	2.95 ±0.82	3.29 ±0.68	3.05 ±0.67	74.85 ±12.34

The highest result (114 points) was obtained by an amateur bodybuilder. Seven persons obtained the result of 111 points – four persons from Group 1 (two judokas, a kickboxer and a taekwondo athlete), two persons from Group 2 (athletes) and one person from Group 3. The person with the lowest result (40) belonged to Group 3. Among the persons physically active the lowest score (51) was obtained by a powerlifter. Moreover, three sportsmen from Group 1 (an amateur judoka and a wrestler and taekwondo athlete) obtained 52 points. Every fifth participant of the study from Group 1 and 2 presented a high level of health behaviours. Only every tenth person in Group 3 achieved a result indicating a high level of health behaviours, and more than a half (59%) obtained a result indicating a very low level (Table 3).

**Table 3:** Categories of health-related behaviours of examined people

***Groups***	***High N(%)***	***Average N(%)***	***Low N(%)***
Group 1 – combat sports	45 (20)	84 (38)	92 (42)
Group 2 – active	30 (19)	55 (34)	75 (47)
Group 3 – non-active	19 (11)	55 (31)	106 (59)

The persons practicing combat sports and martial arts obtained the highest result in the category of psychical attitude (3.5), which was similar to the result of the non-active group (3.29). The men belonging to Group 2 rated highest their eating habits (3.37). All groups obtained the lowest results in the category of preventive behaviours. No significant differences were observed between Group 1 and 2 in terms of eating habits and health practices. Whereas, significant differences were noted in the other categories – preventive behaviours (*P*=0.022) and psychical attitude (*P*=0.003). The men who were not physically active obtained significantly lower results in each category of health behaviours than the combat sports and martial arts competitors (Table 2).

There was a negative correlation observed in Group 1 between age and health practices, as well as a positive correlation between training experience and frequency of trainings and eating habit and preventive behaviours (Table 4).

**Table 4:** Correlations (rho Spearmann) between health-related behaviours and selected features

***Groups***	***Variable***	***Nutrition habits***	***Prophylactic behaviour***	***Positive attitude***	***Healthy practices***	***Sum***
Group 1 – combat sports	Age	−0.049	−0.070	0.105	−0.162*	−0.058
	BMI	−0.052	−0.109	−0.015	−0.118	−0.129
	Training experience	0.152*	0.173**	0.105	−0.062	0.122
Group 2 – active	Frequency of training	0.135*	0.171*	−0.050	−0.055	0.077
	Age	0.025	0.104	0.114	−0.039	0.076
	BMI	0.016	0.008	0.129	−0.105	0.016
	Training experience	−0.037	0.027	−0.042	0.091	0.022
Group 3 – non-active	Frequency of training	0.054	0.182*	0.021	0.085	0.107
	Age	0.118	0.135	0.066	0.004	0.104
	BMI	0.189*	0.023	0.077	−0.034	0.097

* *P*<0.05;

** *P*<0.01

No significant differences were observed both in general level of health behaviours and particular categories in the case of men practicing different disciplines. The kickboxers presented the highest level of health behaviours (84.63), while the lowest level was observed in the case of the men practicing amateur kickboxing (77.26). Competitive kickboxers obtained the highest result in two categories of health behaviours – eating habits and psychical attitude. The highest average result in the category of preventive behaviours was observed among competitive judokas, and in the category of health practices – among amateur judokas. aA high level of health behaviours was noted in the case of from 13% to 27% of persons competing in particular disciplines (the highest number was observed among the competitive kickboxers). More than a half of amateur kick-boxers and wrestlers were classified as persons presenting a low level of health behaviours.

Taking into account the division into subgroups, including age, training experience and frequency of trainings, no significant differences were noted in terms of general health behaviours indicator. The seniors (competitors older than 24 yr) obtained a significantly higher result in terms of psychical attitude, while juniors and youth – in terms of health practices. Moreover, the highest results in the NH (nutrition habits) and PB (preventive behaviours) categories were obtained by the sportsmen training more often than four times a week. Training experience was not a factor differentiating the participants of the study in any category of health behaviours.

## Discussion

Combat sports can be practiced at any age since they aim at a general development and offer a wide variety of exercises, while the persons who practice this kind of sport are not only stronger and more enduring but also have better motor coordination, concentration, balance and flexibility (12–14).

The competitive sportsmen examined in the present study displayed a higher level of health behaviours than the non-training persons, or the persons training other sports disciplines. Regular training of martial arts can contribute to a lifestyle change, help getting rid of unhealthy habits, and influence mentality. For this reason, the elements of martial arts found use in treatment and rehabilitation (15, 16). Positive outcomes were observed as a result of implementing judo training as a supplement of the therapy of children with mental disorders and intellectual disabilities (17, 18). Practicing different forms of combat sports reduces the level of aggression of the person training (19, 20). Practicing martial arts, thanks to their ethical dimension, prevents risky health behaviours. The trainees are less likely to engage in risky health behaviours (21–23). The present study supported that claim by proving that persons practicing martial arts pay more attention to maintaining healthy lifestyle.

Combat sports and martial arts are often described as “high-risk sports”. They are commonly considered to be dangerous for health because of high risk of experiencing a trauma during the training (24–26). However, among persons practicing football, volleyball, gymnastics and martial arts, combat sportsmen were injured least frequently (27). Among the most frequent injuries experienced by combat sportsmen, Wilkerson (28) includes minor injuries of soft tissues, haematomas and lacerations, and, less frequently, fractures. More serious damages are related to high-level sports, where injuries occur also in other disciplines. According to various estimates, from several to less than twenty percent of sportsmen suffer from injuries (29–32). The majority of them concern combat sports. During large sporting events, injuries eliminate from participating in or prevent from completing a contest as many as every eighth contestant (24). During the London Olympics, injuries were reported in the case of 12.9% of sportsmen, the majority of which (54.9%) occurred during tournaments (33). Among the combat sportsmen, only in taekwondo the percentage of the injured was higher than the average for all the other disciplines (39.1%). In the case of wrestlers, judokas, and boxers, fewer injuries were reported than in football, handball, athletics, weightlifting, mountain biking, field hockey, sailing, triathlon, badminton, synchronized swimming, water polo or beach volleyball (33). Similarly, in Paralympic sports, injuries are reported more often in the case of rugby players, athletes or weightlifters than judokas (34). Woodward (15) specifies that combat sports are relatively safe in the comparison with other disciplines, and the majority of combat sportsmen suffer only from minor injuries, especially at the beginner and intermediate levels (35).

Contemporary lifestyle does not favour being physical active. Sedentary work and lack of time for any activities cause that frequently people are obese and less fit, which goes hand in hand with being in higher risk of diseases. The aging population serves a reminder that this problem will continue to grow. It is therefore very important to create an efficient programme of health education and prevention targeted towards promoting physical activeness as well as martial arts as a beneficial form of active recreation and so-called “entire-life sport” (36). Among others, by own research, physically active persons are more often aware of the importance of lifestyle in maintaining good health, which is why they less often smoke, choose healthier diet and more often undergo preventive medical examinations (21, 37, 38). The factors that play an important role is the environment and the coach (trainer), who is, at the same time, an educator and a teacher, and, in Far-Eastern martial arts, also a master (sensei) (7).

Consider the impact on the results could have other environmental factors (disturbing variables), although they occurred in all groups under study. The training of sports and martial arts was the main form of physical activity implemented by the research participants Group 1, so its impact on the studied traits was significant.

The questionnaire survey may raise doubts about the objectification of the answers obtained. Inconsistencies, which present a risk of differing interpretations, were tried on a regular basis, fairly explained. Any individual survey errors do not affect the overall results of the survey.

## Conclusion

The persons practicing combat sports showed high general level of health behaviour indicator – they more often paid attention to proper eating habits and health practices. It may give evidence to the intellectualization of sport and health training process, as well as to the fact of adopting the desired behaviours in everyday life.

It seems reasonable to continue to promote physical activeness in all groups in the society, including combat sports and martial arts as one of its valuable forms.

## Ethical considerations

Ethical issues (Including plagiarism, informed consent, misconduct, data fabrication and/or falsification, double publication and/or submission, redundancy, etc.) have been completely observed by the authors.
